# Synergistic Activity of the Plant Defensin HsAFP1 and Caspofungin against *Candida albicans* Biofilms and Planktonic Cultures

**DOI:** 10.1371/journal.pone.0132701

**Published:** 2015-08-06

**Authors:** Kim Vriens, Tanne L. Cools, Peta J. Harvey, David J. Craik, Pieter Spincemaille, David Cassiman, Annabel Braem, Jozef Vleugels, Peter H. Nibbering, Jan Wouter Drijfhout, Barbara De Coninck, Bruno P. A. Cammue, Karin Thevissen

**Affiliations:** 1 Centre of Microbial and Plant Genetics, KU Leuven, Leuven, Belgium; 2 Institute for Molecular Bioscience, The University of Queensland, Brisbane, Australia; 3 Department of Hepatology, University Hospitals Leuven, Leuven, Belgium; 4 Department of Laboratory Medicine, University Hospitals Leuven, Leuven, Belgium; 5 Metabolic Center, University Hospitals Leuven, Leuven, Belgium; 6 Department of Materials Engineering, KU Leuven, Leuven, Belgium; 7 Department of Infectious Diseases, Leiden University Medical Center, Leiden, The Netherlands; 8 Department of Immunohematology and Bloodtransfusion, Leiden University Medical Center, Leiden, The Netherlands; 9 Department of Plant Systems Biology, VIB, Ghent, Belgium; Hans-Knoell-Institute (HKI), GERMANY

## Abstract

Plant defensins are small, cysteine-rich peptides with antifungal activity against a broad range of yeast and fungi. In this study we investigated the antibiofilm activity of a plant defensin from coral bells (*Heuchera sanguinea*), *i*.*e*. HsAFP1. To this end, HsAFP1 was heterologously produced using *Pichia pastoris* as a host. The recombinant peptide rHsAFP1 showed a similar antifungal activity against the plant pathogen *Fusarium culmorum* as native HsAFP1 purified from seeds. NMR analysis revealed that rHsAFP1 consists of an α-helix and a triple-stranded antiparallel β-sheet stabilised by four intramolecular disulfide bonds. We found that rHsAFP1 can inhibit growth of the human pathogen *Candida albicans* as well as prevent *C*. *albicans* biofilm formation with a BIC50 (*i*.*e*. the minimum rHsAFP1 concentration required to inhibit biofilm formation by 50% as compared to control treatment) of 11.00 ± 1.70 μM. As such, this is the first report of a plant defensin exhibiting inhibitory activity against fungal biofilms. We further analysed the potential of rHsAFP1 to increase the activity of the conventional antimycotics caspofungin and amphotericin B towards *C*. *albicans*. Synergistic effects were observed between rHsAFP1 and these compounds against both planktonic *C*. *albicans* cells and biofilms. Most notably, concentrations of rHsAFP1 as low as 0.53 μM resulted in a synergistic activity with caspofungin against pre-grown *C*. *albicans* biofilms. rHsAFP1 was found non-toxic towards human HepG2 cells up to 40 μM, thereby supporting the lack of a general cytotoxic activity as previously reported for HsAFP1. A structure-function study with 24-mer synthetic peptides spanning the entire HsAFP1 sequence revealed the importance of the γ-core and its adjacent regions for HsAFP1 antibiofilm activity. These findings point towards broad applications of rHsAFP1 and its derivatives in the field of antifungal and antibiofilm drug development.

## Introduction

Plant defensins are small, basic, cysteine-rich peptides with a conserved structure known as a cysteine-stabilized αβ-motif [1–3]. Although the tertiary structure of some plant defensins [2,4–7] is known, the structure of many defensins is yet to be determined. Plant defensins exhibit antimicrobial activity against a wide range of microorganisms [8–10], whereas they are in general non-toxic to human cells [11–13]. To date, there has been a particular focus on their antifungal activity and several fungal targets have been identified, including membrane sphingolipids and phospholipids [14–20]. Upon interaction with the fungal membrane, plant defensins are either internalized into the cell and interact with cytosolic or nuclear proteins, or they remain localized at the cell wall or membrane of the fungus [4,21–23]. The mechanisms by which plant defensins induce fungal cell death are diverse, but common aspects are observed. These include the production of reactive oxygen species (ROS) and the induction of apoptosis [24].

Despite the fact that their mechanisms of antifungal action have been studied extensively, no reports exist about the activity of plant defensins against fungal biofilms. Biofilms are self-organised microbial communities embedded in a polymeric matrix that grow on a biotic or abiotic surface, such as catheters or other medical implants. Many fungal species are able to form biofilms, however, *Candida* spp. play a predominant role in mixed-species fungal biofilms [25–28]. Such biofilm cells are tolerant towards most conventional antimycotics and there are only few novel agents that can be used to treat biofilm-related infections. To date, only miconazole, caspofungin, anidulafungin and liposomal formulations of amphotericin B are used to effectively treat these infections [29–31], and hence, there is a need to identify novel antibiofilm compounds.

In this study, we used the defensin from coral bells, HsAFP1, which was previously characterized by Osborn and colleagues [32], and assessed its potential antibiofilm activity. HsAFP1 inhibits the growth of various plant pathogenic fungi, including *Botrytis cinerea*, *Verticillium albo-atrum* and *Fusarium culmorum*, and causes swelling of germ tubes and hyphae in the latter [32]. In addition, it was reported that HsAFP1 shows antifungal activity against *Saccharomyces cerevisiae* and the human pathogen *C*. *albicans*, and induces apoptosis in the latter [33]. Furthermore, it was shown that HsAFP1 has a low *in vitro* frequency of resistance occurrence in planktonic *C*. *albicans* cultures (*i*.*e*. less than 1 in 2,000,000 mutants) [11]. In an attempt to unravel HsAFP1’s mode of antifungal activity, this defensin was tested against the complete *S*. *cerevisiae* deletion mutant library for identification of yeast mutants with altered HsAFP1 sensitivity [33]. In this study, 84 yeast genes were identified that were found to be implicated in governing HsAFP1 tolerance or sensitivity of yeast [33]. Since HsAFP1 has a potent antifungal activity towards *C*. *albicans*, we further analysed its potential activity towards *C*. *albicans* biofilms. To this end, we heterologously expressed HsAFP1 using the yeast *Pichia pastoris* and determined the solution structure of recombinant (r) rHsAFP1 by NMR analysis. Subsequently, we tested the activity of the plant defensin alone and in combination with conventional antimycotics against *C*. *albicans* biofilms. In view of the latter, a multi-drug approach in which multiple compounds are administered and a synergistic effect is observed, can be effectively used to combat biofilm-related infections [34]. Finally, we conducted a structure-function study, using 24-mer synthetic peptides spanning the entire HsAFP1 region. The HsAFP1 derivatives were tested against *C*. *albicans* planktonic cultures and biofilms, and their potential to synergistically enhance the activity of caspofungin was analysed.

## Materials and Methods

### Strains and reagents

*Pichia pastoris* strain X33 was used for heterologous production of HsAFP1. *Fusarium culmorum* strain K0311 was used to evaluate the antifungal activity of the recombinant peptide and to compare it with that of native HsAFP1 purified from seeds, in a fungal growth inhibitory assay [32]. *C*. *albicans* strain SC5314 was used in all biofilm experiments. rHsAFP1 toxicity testing was performed on HepG2, human hepatoma cells [35], purchased from ATCC (catalogue number HB-8065; Rockville, MD, USA).

All culture media were purchased from LabM (UK), unless stated otherwise. For heterologous production, *P*. *pastoris* was cultured in YPD (1% yeast extract, 2% peptone and 2% glucose), BMGY (buffered complex glycerol medium; 1% yeast extract, 2% peptone, 1.34% yeast nitrogen base w/o amino acids (Becton Dickinson, UK), 1% glycerol, 100 mM K_3_PO_4_ pH 6, 4 x 10^−5^% biotin) or BMMY (buffered complex methanol medium; 1% yeast extract, 2% peptone, 1.34% yeast nitrogen base w/o amino acids (Becton Dickinson, UK), 0.5% methanol, 100 mM K_3_PO_4_ pH 6, 4 x 10^−5^% biotin). *F*. *culmorum* was grown in half strength PDB (1.2% potato dextrose broth). Biofilm experiments were performed in RPMI-1640 medium (Roswell Park Memorial Institute-1640 medium; pH 7) with L-glutamine and without sodium bicarbonate (purchased from Sigma Aldrich, St.-Louis, MO, USA), buffered with MOPS (Sigma Aldrich, St.-Louis, MO, USA). Amphotericin B and caspofungin (Cancidas) were purchased from Sigma Aldrich (St. Louis, MO, USA) and Merck (Beeston Nottingham, UK), respectively. HepG2 cells were grown in MEM (Minimal Essential Medium, Gibco, Invitrogen; CA, USA), supplemented with 10% fetal calf serum, 2 mM L-glutamine, 100 U/mL penicillin and 100 μg/mL streptomycin, and cultured using standard cell culture conditions (37°C, 5% CO_2_, 95% humidity). The Cell Proliferation Kit II (XTT) and Cell Proliferation ELISA BrdU (colorimetric) kit were purchased from Roche Diagnostics (Mannheim, Germany).

### Production and purification of recombinant (r) rHsAFP1

The PCR fragment encoding mature HsAFP1 was cloned in frame with the α-factor secretion signal present in the pPICZαA transfer vector, after which the plasmid was integrated into the genome of *Pichia pastoris* X33 strain via double homologous recombination. This transgenic *P*. *pastoris* strain was grown in YPD overnight at 30°C and 250 rpm. BMGY medium was inoculated with the overnight culture to an optical density at 600 nm (OD_600nm_) of 0.5 and grown for 24 hours at 30°C and 200 rpm. Cells were pelleted by sterile centrifugation at 8000 rpm for 10 minutes at room temperature and re-suspended in BMMY medium, thereby concentrating the culture 4-fold and inducing gene expression. The culture was grown for 96 hours at 25°C, and 2.5% methanol (v/v%) was added to the culture every 24 hours to maintain induction of gene expression. After induction, cells were pelleted at 8000 rpm for 10 minutes at 4°C and the cleared supernatant, containing the peptides of interest, was filter sterilized through a Steritop-GP 0.22 μm Express PLUS membrane Bottle-top filter (EMD Millipore, MA, USA). The filtered supernatant was then subjected to automated tangential flow filtration using an automated peristaltic pump (Spectrum Laboratories, CA, USA) and a hollow fiber module with 1 kDa cut-off mPES membranes (Spectrum Laboratories, CA, USA). During the ultrafiltration, the sample was concentrated a 15-fold and subsequently dialyzed against 50 mM sodium acetate pH 5.

rHsAFP1 was purified by cation exchange chromatography, using 75 mL SP sepharose High Performance resin (GE Healthcare, UK) packed in a XK26/20 column (GE Healthcare) and 50 mM sodium acetate buffers at pH 5. The flow rate was maintained at 5 mL/min. Elution of the peptides was carried out by a washing step with 10% (v/v%) elution buffer (50 mM sodium acetate, 1 M sodium chloride, pH 5) for 10 column volumes (CV), followed by a linear gradient to 50% (v/v%) elution buffer in 15 CV, resulting in a peak at approximately 29% (v/v%) elution buffer. The eluted fraction was further purified by reversed phase chromatography employing a Gemini C18 250x10 column (Phenomenex, CA, USA) and acetonitrile (ACN) for elution of the bound peptides. The flow rate was maintained at 4.6 mL/min. Elution of the peptides was carried out by a washing step at 15% (v/v%) ACN for 1.9 CV, followed by a linear gradient to 35% (v/v%) ACN in 2.3 CV. Elution of rHsAFP1 occurred at 28%. The eluted fraction was vacuum dried by centrifugal evaporation (SpeedVac Savant, Thermo Fisher Scientific, MA, USA), re-dissolved in MilliQ water and subjected to a micro bicinchoninic acid assay (Pierce, Thermo Scientific, USA) according to the manufacturer’s instructions, to determine the protein concentration. Bovine serum albumin served as a reference protein. At least 40 mg/L of culture of purified rHsAFP1 was obtained.

### Characterization of rHsAFP1 by NMR

Dry powder (1 mg) of rHsAFP1 was dissolved in 500 μL of 10% D_2_O/90% H_2_O (~pH 4) for NMR experiments. Spectra were recorded at 298 K on a Bruker Avance-600 spectrometer. Two-dimensional NMR experiments included total correlation spectroscopy (TOCSY [36]) using a MLEV-17 spin lock sequence [37] with a mixing time of 80 ms; nuclear Overhauser effect spectroscopy (NOESY [38]) with a mixing time of 150, 200, or 300 ms; exclusive correlation spectroscopy (ECOSY [39]); and ^13^C and ^15^N heteronuclear single-quantum coherence (HSQC [40]). Solvent suppression was achieved using excitation sculpting with gradients [41]. Spectra were acquired with 4096 complex data points in F2 and 512 increments in the F1 dimension. Slowly exchanging amide protons were identified by spectra also recorded in 100% D_2_O.

Spectra were processed using TopSpin (Bruker) software. The *t*1 dimension was zero-filled to 1024 real data points, and 90° phase-shifted sine bell window functions were applied prior to Fourier transformation. Chemical shifts were referenced to internal 2,2-dimethyl-2-silapentane-5-sulfonate (DSS). Processed spectra were analysed and assigned using CcpNmr Analysis [42]. Spectra were assigned using the sequential assignment protocol [43].

### Structure calculations

Structure calculations were based on distance restraints derived from NOESY spectra recorded in both 10% and 100% D_2_O. Initial structures were generated using the program CYANA [44], followed by addition of restraints for the disulfide bonds, hydrogen bonds as indicated by slow D_2_O exchange and sensitivity of amide proton chemical shift to temperature, chi1 restraints from ECOSY and NOESY data, and backbone phi and psi dihedral angles restraints generated using the program TALOS+ [45]. The structural family was generated using torsion angle dynamics, refinement and energy minimization in explicit solvent and protocols as developed for the RECOORD database [46] within the program CNS [47]. A family of structures consistent with the experimental restraints was then visualized using MOLMOL [48] and assessed for stereochemical quality using MolProbity [49]. Coordinates and NMR chemical shift assignments have been submitted (PDB ID: 2n2q; BMRB ID: 25605).

### Antifungal activity assays

To test whether rHsAFP1 is as potent as HsAFP1 purified from the seeds of coral bells, we analysed the antifungal activity of both peptides against *F*. *culmorum*, following the standard CLSI protocol M28-A2 [50], with minor modifications as previously described by Osborn and colleagues [32]: an inoculum of approximately 10^4^ spores/mL of *F*. *culmorum* was suspended in half strength PDB and added to a two-fold dilution series of rHsAFP1 in water. Seed-derived HsAFP1 was purified according to the protocol as previously described by Osborn and colleagues [32]. The IC50 value, which is the concentration required for 50% growth inhibition as compared to control treatment, was determined by measuring the optical density at 490 nm (OD_490nm_) after 48 hours of incubation and was confirmed microscopically. The antifungal activity of rHsAFP1 against *C*. *albicans* was subsequently analysed according to the standard CLSI protocol M27-A3 [51] with minor modifications: an inoculum of approximately 10^6^ cells/mL was suspended in RPMI-1640 medium and added to a two-fold dilution series of rHsAFP1 in water. The DMSO concentration was similar to that in the biofilm assays, *i*.*e*. 0.5% DMSO. The MIC50 value, *i*.*e*. the minimum concentration required to reduce planktonic growth by 50% as compared to control treatment, was determined by measuring the OD_490nm_ after 24 hours of incubation.

### Antibiofilm activity assays

#### Biofilm inhibition assay

The Biofilm Inhibitory Concentration 50 value (BIC50; the minimum concentration required to reduce biofilm formation by 50% as compared to control treatment) of rHsAFP1 was determined using the following antibiofilm assay: a *C*. *albicans* SC5314 overnight culture, grown in YPD, was diluted to an optical density (600 nm) of 0.1 in RPMI 1640 medium and 100 μL of this suspension was added to the wells of a round-bottomed microtitre plate (TPP, Tradingen, Switzerland). After 1 h of adhesion at 37°C, the medium was aspirated and the biofilms were washed with 100 μL phosphate-buffered saline (PBS) to remove non-adherent cells. Fresh RPMI 1640 medium, followed by an rHsAFP1 concentration series was added to the biofilms. The DMSO concentration was similar to that in the checkerboard assays, *i*.*e*. 0.5%. Biofilms were allowed to grow for 24 h at 37°C and were subsequently washed with PBS and quantified with CellTiter-Blue (CTB; Promega, WI, USA)) [52] by adding 100 μL of CTB diluted 1/10 in PBS to each well. After 1 h of incubation in the dark at 37°C, the fluorescence was measured with a fluorescence spectrometer (λ_Ex_/λ_Em_: 535/590 nm). The fluorescence values of the samples were corrected by subtracting the average fluorescence value of the CTB of uninoculated wells (blank). The percentage of surviving biofilm cells was calculated relative to the control treatment (0.5% DMSO).

#### Biofilm eradication assay

The Biofilm Eradicating Concentration 50 value (BEC50; the minimum concentration required to reduce the viability of the cells in a pre-grown biofilm by 50% as compared to control treatment) of rHsAFP1 was determined using the BEC50 determination assay as described by De Cremer and co-workers [53]. Briefly, a *C*. *albicans* SC5314 overnight culture, grown in YPD, was diluted to an optical density (600 nm) of 0.1 in RPMI 1640 medium and 100 μL of this suspension was added to the wells of a round-bottomed microtitre plate (TPP, Tradingen, Switzerland). After 1 h of adhesion, the biofilms were washed with 100 μL PBS to remove non-adherent cells, followed by addition of 100 μL RPMI 1640 medium. The biofilms were allowed to grow for 24 h at 37°C. Next, an rHsAFP1 concentration series in RPMI was added to the biofilms. The DMSO concentration was similar to that in the checkerboard assays, *i*.*e*. 0.5%. The biofilms were incubated for another 24 h at 37°C, after which they were washed and quantified with CTB as described above.

#### Checkerboard assay

*C*. *albicans* biofilms or *C*. *albicans* planktonic cultures were grown as described above. A combination of rHsAFP1 and antimycotic (caspofungin or amphotericin B), two-fold diluted across the columns and rows of a 96-well plate, respectively, was added to the planktonic culture or to the biofilms. Biofilms were treated either after 1 hour or 24 hours starting from the adhesion phase to analyse biofilm inhibition or biofilm eradication, respectively. After 24 hours incubation at 37°C, the MIC50 values were determined by measuring the OD_490nm_, whereas BIC50 and BEC50 values were determined using CTB as described above. In all experiments, the DMSO concentration was kept at 0.5%. Synergy was determined by calculating the Fractional Inhibitory Concentration Index (FICI) [54,55], in which the actual concentration of compound A (*i*.*e*. rHsAFP1) in the checkerboard experiment was used, as indicated in Tables 1–3 and 4.

**Table 1:** Synergistic activity of rHsAFP1 with caspofungin or amphotericin B against *C*. *albicans* SC5314 biofilms, resulting in biofilm formation inhibition*.

Compound(s)	[rHsAFP1] (μM)	BIC50 CAS or AMB (μM) ± SEM	Fold change	FICI	Significance level
CAS alone	0	0.72 ± 0.05	NA	NA	
CAS + rHsAFP1	8.4	0.05 ± 0.00	15.8	0.86	***
	4.2	0.10 ± 0.01	7.1	0.54	***
	**2.1**	**0.20 ± 0.01**	**3.7**	**0.47**	
	**1.05**	**0.28 ± 0.04**	**2.5**	**0.49**	
	0.53	0.42 ± 0.02	1.7	0.64	**
	0.26	0.65 ± 0.07	1.1	0.93	NS
AMB alone	0	1.23 ± 0.15	NA	NA	
AMB + rHsAFP1	8.4	0.60 ± 0.07	2.1	1.28	**
	4.2	0.53 ± 0.05	2.3	0.83	**
	2.1	0.56 ± 0.04	2.2	0.65	**
	1.05	0.67 ± 0.06	1.8	0.64	*
	0.53	0.81 ± 0.11	1.5	0.71	NS
	0.26	0.93 ± 0.11	1.3	0.78	NS

* BIC50 values were determined by CTB assay; mean ± SEM for n ≥ 3 independent experiments is presented; BIC50, minimum inhibitory concentration that is required to inhibit biofilm formation by 50% as compared to control treatment; FICI, Fractional Inhibitory Concentration Index, FICI ≤ 0.5 indicates synergy between two compounds; NA, not applicable; CAS, caspofungin; AMB, amphotericin B. Values in bold represent synergistic effects between two compounds. Unpaired Student t-tests were performed in case FICI did not indicate synergy to analyse significant differences between the effect of the compound alone and the combination of compound and rHsAFP1; the significance level is presented (*, ** and *** represent *P*<0.05, *P*<0.01 and *P*<0.001, respectively; NS, no significant difference).

**Table 2:** Synergistic activity of rHsAFP1 with caspofungin or amphotericin B against *C*. *albicans* SC5314 biofilms, resulting in eradication of *C*. *albicans* biofilm cells*.

Compound(s)	[rHsAFP1] (μM)	BEC50 CAS or AMB (μM) ± SEM	Fold change	FICI (<)	Significance level
CAS alone	0	0.40 ± 0.08	NA	NA	
CAS + rHsAFP1	**16.8**	**0.04 ± 0.01**	**9.7**	**0.26**	
	**8.4**	**0.06 ± 0.00**	**7.2**	**0.22**	
	**4.2**	**0.07 ± 0.00**	**5.7**	**0.21**	
	**2.1**	**0.10 ± 0.01**	**4.0**	**0.27**	
	**1.05**	**0.12 ± 0.01**	**3.3**	**0.32**	
	**0.53**	**0.15 ± 0.02**	**2.7**	**0.37**	
AMB alone	0	1.67 ± 0.30	NA	NA	
AMB + rHsAFP1	16.8	0.61 ± 0.17	2.7	0.52	*
	**8.4**	**0.66 ± 0.22**	**2.5**	**0.47**	
	4.2	0.82 ± 0.19	2.1	0.53	*
	2.1	0.95 ± 0.28	1.8	0.59	NS
	1.05	1.07 ± 0.29	1.6	0.65	NS
	0.53	1.28 ± 0.28	1.3	0.77	NS

* BEC50 values were determined by CTB assay; mean ± SEM for n ≥ 3 independent experiments is presented; BEC50, minimum concentration that is required to reduce viability of 24 hours-old biofilm cells by 50% as compared to control treatment; FICI, Fractional Inhibitory Concentration Index, FICI ≤ 0.5 indicates synergy between two compounds; NA, not applicable; CAS, caspofungin; AMB, amphotericin B. Values in bold represent synergistic effects between two compounds. Unpaired Student t-tests were performed in case FICI did not indicate synergy to analyse significant differences between the effect of the compound alone and the combination of compound and rHsAFP1; the significance level is presented (*, ** and *** represent *P*<0.05, *P*<0.01 and *P*<0.001, respectively; NS, no significant difference).

**Table 3:** Synergistic activity of rHsAFP1 with caspofungin or amphotericin B against *C*. *albicans* SC5314 planktonic cultures*.

Compound(s)	[rHsAFP1] (μM)	MIC50 CAS or AMB (μM) ± SEM	Fold change	FICI	Significance level
CAS alone	0	0.02 ± 0.01	NA	NA	
CAS + rHsAFP1	8.4	0.01 ± 0.00	8.6	0.58	NS
	**4.2**	**0.01 ± 0.00**	**4.0**	**0.48**	
	**2.1**	**0.01 ± 0.00**	**2.6**	**0.50**	
	1.05	0.01 ± 0.00	1.8	0.63	NS
	0.53	0.02 ± 0.00	1.2	0.86	NS
	0.26	0.02 ± 0.01	1.1	0.90	NS
AMB alone	0	0.44 ± 0.06	NA	NA	
AMB + rHsAFP1	8.4	0.14 ± 0.01	3.2	0.78	**
	4.2	0.15 ± 0.01	3.0	0.57	**
	**2.1**	**0.15 ± 0.01**	**2.9**	**0.47**	
	**1.05**	**0.17 ± 0.01**	**2.6**	**0.44**	
	**0.53**	**0.18 ± 0.02**	**2.4**	**0.44**	
	0.26	0.25 ± 0.01	1.8	0.58	*

* MIC50 values were determined by measuring the OD at 490 nm; mean ± SEM for n ≥ 3 independent experiments is presented; MIC50, minimum inhibitory concentration that is required to reduce planktonic growth by 50% as compared to control treatment; FICI, Fractional Inhibitory Concentration Index, FICI ≤ 0.5 indicates synergy between two compounds; NA, not applicable; CAS, caspofungin; AMB, amphotericin B. Values in bold represent synergistic effects between two compounds. Unpaired Student t-tests were performed in case FICI did not indicate synergy to analyse significant differences between the effect of the compound alone and the combination of compound and rHsAFP1; the significance level is presented (* and ** represent *P*<0.05 and *P*<0.01, respectively; NS, no significant difference).

**Table 4:** Synergistic activity of HsLin06 with caspofungin against *C*. *albicans* SC5314 biofilms, resulting in biofilm formation inhibition*.

Compound(s)	[HsLin06] (μM)	BIC50 CAS (μM) ± SEM	Fold change	FICI	Significance level
CAS alone	0	0.90 ± 0.05	NA	NA	
CAS + HsLin06	175	0.04 ± 0.00	25.67	16.25	***
	87.5	0.03 ± 0.00	27.87	8.14	**
	43.75	0.07 ± 0.01	14.27	4.13	***
	21.88	0.07 ± 0.01	13.50	2.11	***
	10.94	0.07 ± 0.02	13.67	1.09	***
	5.47	0.07 ± 0.02	13.03	0.59	***
	**1.5**	**0.13 ± 0.00**	**7.76**	**0.28**	
	**0.75**	**0.22 ± 0.01**	**4.45**	**0.31**	
	0.38	0.48 ± 0.04	2.04	0.57	*
	0.19	0.68 ± 0.09	1.43	0.78	NS
	0.09	1.05 ± 0.10	0.93	1.18	NS
	0.05	0.93 ± 0.05	1.05	1.05	NS

* BIC50 values were determined by CTB assay; mean ± SEM for n ≥ 3 independent experiments is presented; BIC50, minimum inhibitory concentration that is required to inhibit biofilm formation by 50% as compared to control treatment; FICI, Fractional Inhibitory Concentration Index, FICI ≤ 0.5 indicates synergy between two compounds; NA, not applicable; CAS, caspofungin. Values in bold represent synergistic effects between two compounds. Unpaired Student t-tests were performed in case FICI did not indicate synergy to analyse significant differences between the effect of the compound alone and the combination of compound and rHsAFP1; the significance level is presented (*, ** and *** represent *P*<0.05, *P*<0.01 and *P*<0.001, respectively; NS, no significant difference).

### Scanning electron microscopy (SEM)

Qualitative analysis of samples was performed using scanning electron microscopy (XL30-FEG, FEI). Samples were prepared using a protocol previously described [56]. Briefly, the biofilm-containing titanium discs were rinsed in PBS and fixed in gluteraldehyde (2.5% v/v in a cacodylate buffer). Samples were rinsed three times in PBS, and subsequently dehydrated in a series of ethanol/H_2_O solutions with increasing alcohol content, followed by air drying. Finally, a thin conductive Au-Pd film was sputtered (Edwards S150) on the samples and SEM was operated at standard high-vacuum settings and using 10 mm working distance and 20 keV accelerating voltage.

### rHsAFP1 toxicity in HepG2 cells

HepG2 cells were seeded at 10.000 cells/well in 96 well-plates and incubated for 24 hours. Subsequently, cells were treated with water (untreated) or rHsAFP1 (0.01 μM–40 μM) for 24 hours after which cell viability or cell proliferation was determined using the “Cell Proliferation Kit II (XTT)”, as described previously [57], or the “Cell Proliferation ELISA BrdU (colorimetric) kit”, according to the manufacturer’s instructions, respectively.

### Structure-function analysis of HsAFP1

Synthesis and purification of the 24-mer peptides (HsLin01-HsLin06) spanning the HsAFP1 amino acid sequence was performed as described previously [58]. Cysteine residues were replaced by α-aminobutyric acid to avoid formation of disulfide bonds.

### Data analysis

Data were analysed with GraphPad Prism (GraphPad Software, Inc., CA, USA). For dose-response data, sigmoidal curves were generated using nonlinear regression. The concentration required to cause 50% planktonic growth inhibition (IC50 or MIC50), reduction of biofilm formation (BIC50) and biofilm eradication (BEC50) as compared to control treatment was derived from the whole dose-response curves. In all experiments, mean ± standard error of the mean (SEM) for n ≥ 3 is presented. Unpaired Student t-tests were performed to analyse significant differences between the IC50 value of native HsAFP1 and that of recombinant HsAFP1, and between the MIC50, BIC50 and BEC50 of caspofungin or amphotericin B alone and the combination of these compounds with rHsAFP1 or its derivatives in the checkerboard assays. To analyse significant differences in cell viability or cell proliferation between untreated and rHsAFP1-treated HepG2 cells in the rHsAFP1 toxicity assays, unpaired Student t-tests were performed. In all cases, *P*<0.05 was defined as statistically significant.

## Results

### rHsAFP1 shows potent antifungal activity against filamentous fungi

rHsAFP1 was produced in *Pichia pastoris* and subsequently purified using cation exchange and reversed phase chromatography. A yield of at least 40 mg/L of culture of purified rHsAFP1 was obtained. The antifungal activity of HsAFP1 against a broad range of fungi, including the fungus *Fusarium culmorum*, has been reported previously [32]. In this respect, Osborn and colleagues showed that native HsAFP1 can inhibit growth of *F*. *culmorum* with an IC50 value of 1 μg/mL [32]. Hence, to assess the potency of rHsAFP1, we tested the antifungal activity of rHsAFP1 and native HsAFP1 against *F*. *culmorum* according to the method of Osborn [32]. We found the IC50 values of the recombinant and native peptide against *F*. *culmorum* not to be significantly different, *i*.*e*. 0.45 ± 0.13 μM and 0.23 ± 0.02 μM respectively, with a *P*-value of 0.1707, and hence, rHsAFP1 seems as potent as native HsAFP1.

### Characterization of rHsAFP1 by NMR

**Fig 1:**
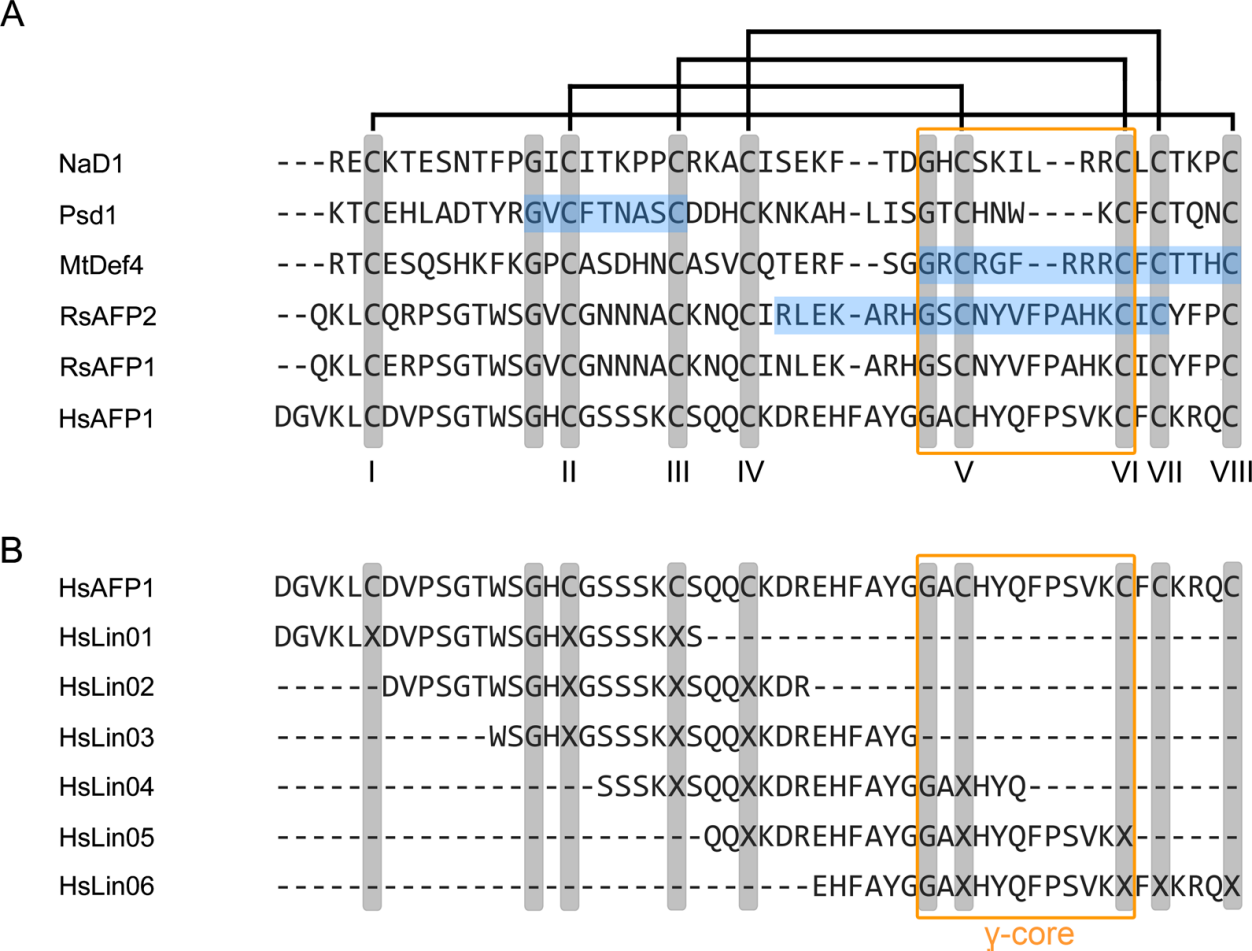
Sequence alignment of HsAFP1 with other plant defensins. (**A**) Amino acid sequence alignment of NaD1 [6], Psd1 [5], MtDef4 [61], RsAFP1 [62], RsAFP2 [62] and HsAFP1 [32], matching their cysteine residues (numbered I-VIII). Multiple alignment was performed using the COBALT alignment tool [63]. Cysteine-pairing is shown at the top of the figure. Highly conserved residues are shown in grey; (-) denote gaps in the alignment. Blue boxes represent peptide fragments that exhibit antifungal activity similar to the parental peptide, and hence, are important for antifungal activity [4,64–66]. The orange box indicates the position of the γ-core. (**B**) Amino acid sequence alignment of HsAFP1 and the HsAFP1 linear peptide fragments (HsLin01-HsLin06). Multiple alignment was performed using the COBALT alignment tool [63]. Highly conserved residues are shown in grey; (-) denote gaps in the alignment. The orange box indicates the position of the γ-core.

The solution structure of rHsAFP1 was solved via NMR analysis, a technique that has been previously used to characterize the structures of other plant defensins, including RsAFP1, MtDef4, Psd1 and NaD1 [2,4–6]. A sequence alignment of HsAFP1 with these peptides and RsAFP2 is presented in Fig 1A, showing the disulfide bond pattern common for plant defensins [59,60].

**Fig 2:**
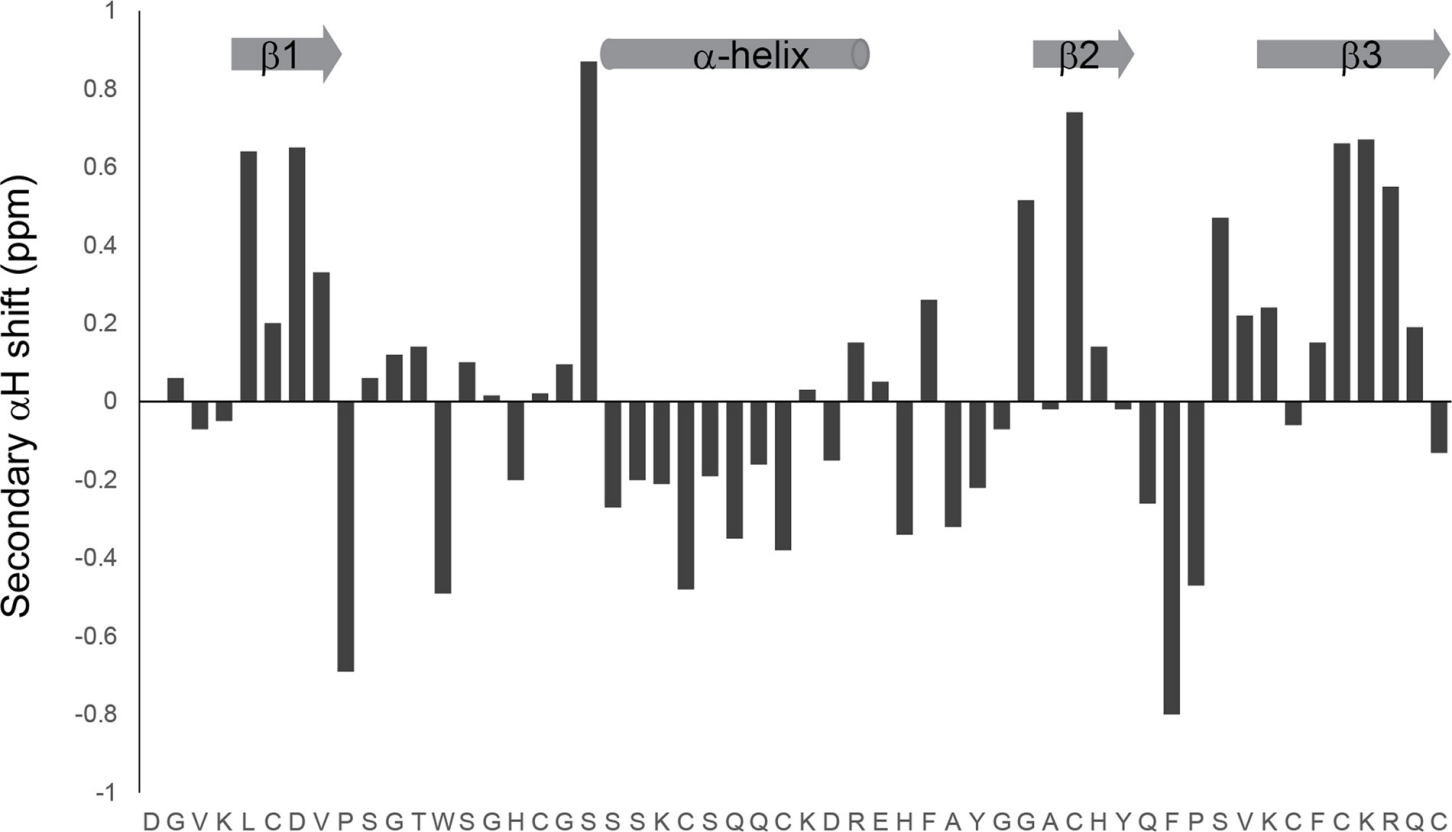
Secondary shift analysis of rHsAFP1, pH 4.0 at 298 K. Regions of α-helix and β-strand are indicated at the top of the figure.

The NMR spectra of rHsAFP1 showed the sample to be of high purity and good dispersion in the amide region was indicative of a highly structured peptide. Two-dimensional spectra were recorded at several temperatures in the range 283 to 303 K to obtain full proton assignments. The proton assignments for rHsAFP1 are presented in S1 Table. Secondary chemical shift analysis was then used to locate elements of secondary structure. Hα secondary shifts are calculated by subtracting the chemical shift of the alpha proton from “random coil” values [67]. Deviations greater than 0.1 ppm from random coil are indicative of structured peptides, with positive values present for beta type structures and negative values for helical structures. The secondary Hα shifts of rHsAFP1 are shown in Fig 2 and indicate that the solution structure of rHsAFP1 consists of both α-helix and β-strand elements.

**Fig 3:**
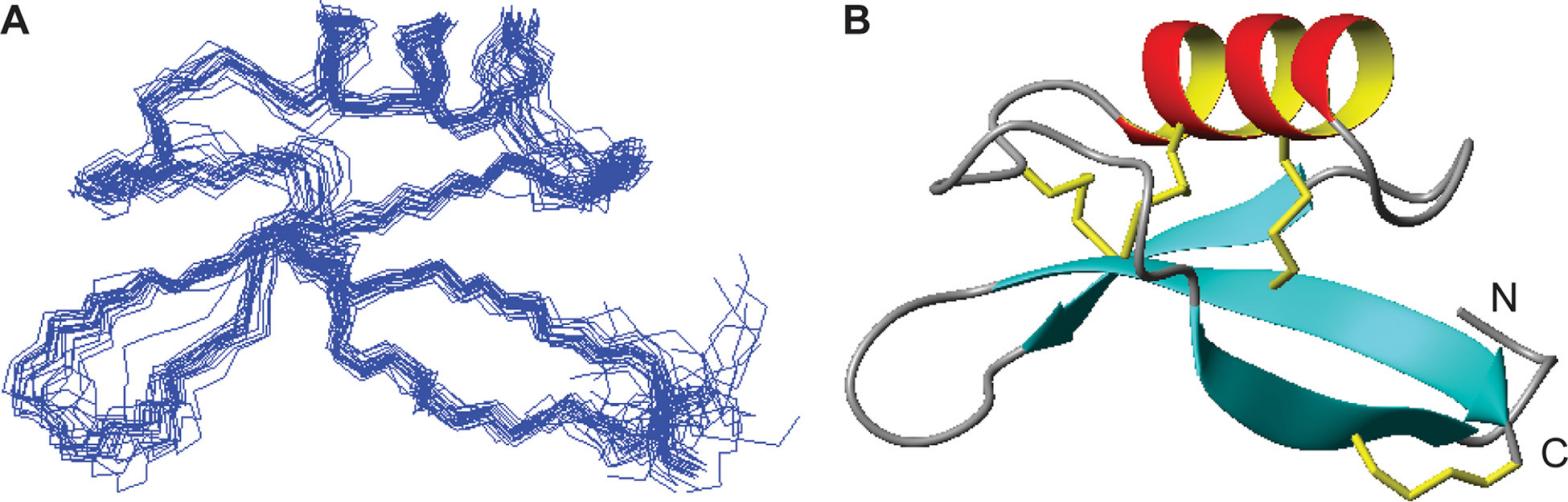
Three-dimensional structure of rHsAFP1. (**A**) A family of 20 lowest energy structures superimposed over all backbone heavy atoms; (**B**) A ribbon representation with disulfide bonds shown in yellow. The termini are labeled as N and C. Diagrams were generated using MOLMOL.

The three-dimensional structure of rHsAFP1 was calculated from 614 distance restraints, 15 hydrogen bond pairs, and a total of 90 dihedral angle restraints (S2 Table). The disulfide connectivities (I-VIII, II-V, III-VI, IV-VII) were fully consistent with the NOE data and were included as restraints in the structure calculations. Similarly to RsAFP1 [2], one proline (Pro9) is present in the *trans* configuration and the second (Pro44) has a *cis* peptide bond. Fig 3A shows the ensemble of structures superimposed over the backbone heavy-atoms of all residues (rmsd 1.16 ± 0.40 Å). A ribbon representation of the lowest energy structure is shown in Fig 3B. Analysis of the structures shows that 96% of residues fall in the most favored regions of the Ramachandran plot and a mean MolProbity score of 1.8 indicates good structural quality. rHsAFP1 forms a compact globular fold with a three turn α-helix spanning residues Ser20-Arg30 and a triple-stranded anti-parallel β-sheet (β1 = Leu5-Pro9; β2 = Ala38-His40; β3 = Lys47-Gln53) forming another element of secondary structure. The four disulfide bonds are arranged in a typical cysteine-stabilized αβ motif in that the α-helix is tethered to the β-sheet by two disulfide bonds to the central strand (Cys23-Cys39 and Cys27-Cys50). There are three loops present in the molecule that link β-strand 1 with the helix, the helix to β-strand 2, and the β-strands 2 and 3. These loops are reasonably well-defined although the loop that incorporates a β-turn between strand 2 and 3 is apparently more flexible as judged by greater disorder in the structural ensemble in this region.

### rHsAFP1 prevents *C*. *albicans* biofilm formation

At first, we assessed the antifungal activity of rHsAFP1 against planktonic *C*. *albicans* cells. rHsAFP1 showed antifungal activity against planktonic *C*. *albicans* cultures, with a MIC50 value of 18.00 ± 4.60 μM. Subsequently, we investigated the ability of rHsAFP1 to prevent or eradicate *C*. *albicans* biofilms. rHsAFP1 inhibited *C*. *albicans* biofilm formation, resulting in a BIC50 value of 11.00 ± 1.70 μM. Fifty percent eradication of *C*. *albicans* biofilms by this peptide, as compared to control treatment, was not observed at the highest tested concentration, *i*.*e*. 109.00 μM (*i*.*e*. BEC50 of rHsAFP1 is > 109.00 μM) (data not shown).

**Fig 4:**
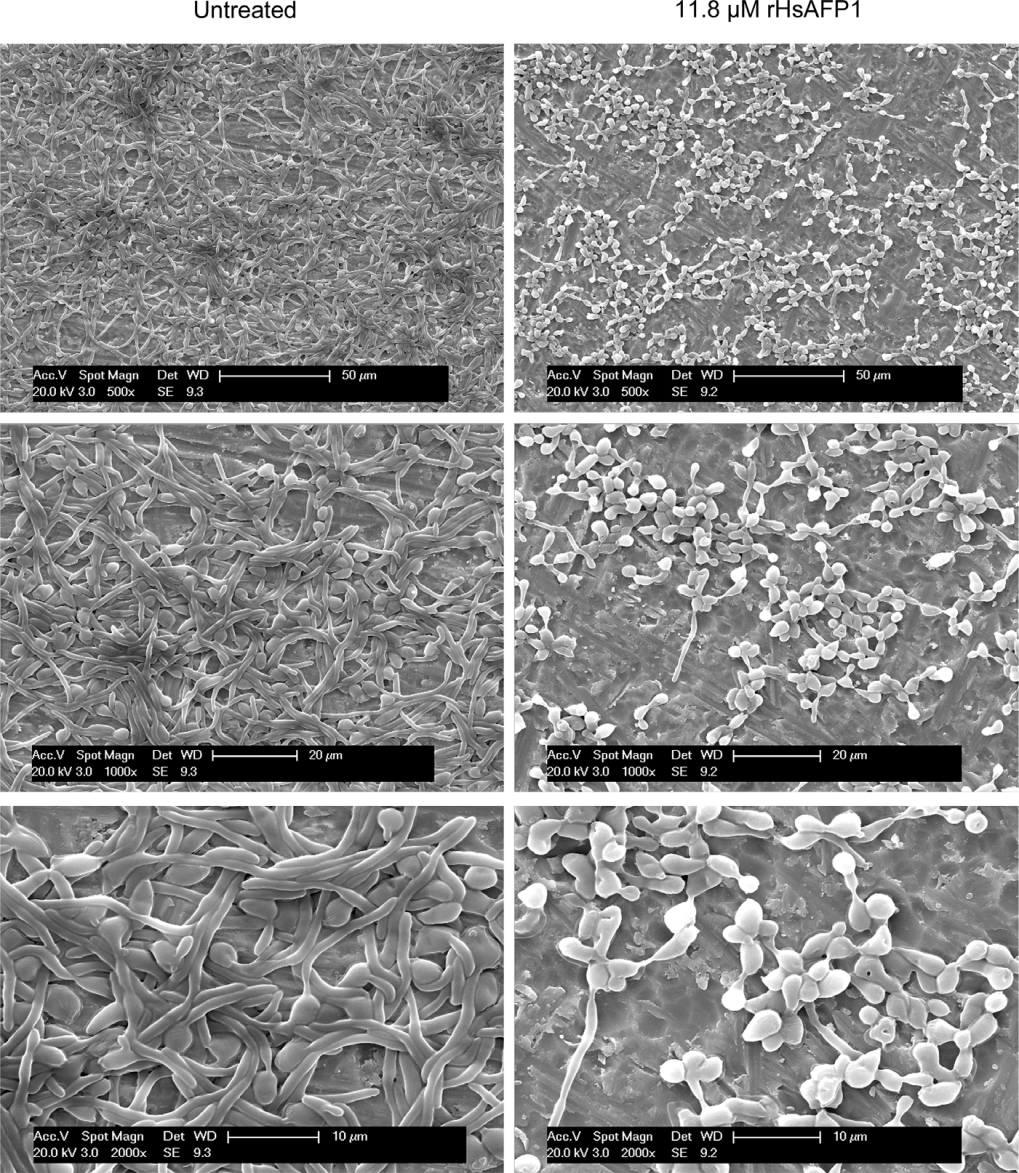
Scanning electron microscopy images of 4 hours-old biofilms, grown in the presence or absence (untreated) of 11.8 μM rHsAFP1. Images at multiple magnifications (500x, 1000x and 2000x) are presented.

In order to investigate the effect of rHsAFP1 on the growth of *C*. *albicans* biofilms, SEM images of biofilms grown for 4 hours in the presence or absence of rHsAFP1 (11.8 μM) were taken. As shown in Fig 4, cells in the untreated biofilms were able to form a dense hyphal network, covering the titanium discs. In contrast, no true biofilm was formed in the presence of 11.8 μM rHsAFP1, as in this case, biofilms mainly consisted of cells attached to the titanium disc without formation of a hyphal network.

### rHsAFP1 acts synergistically with caspofungin or amphotericin B against *C*. *albicans*

**Fig 5:**
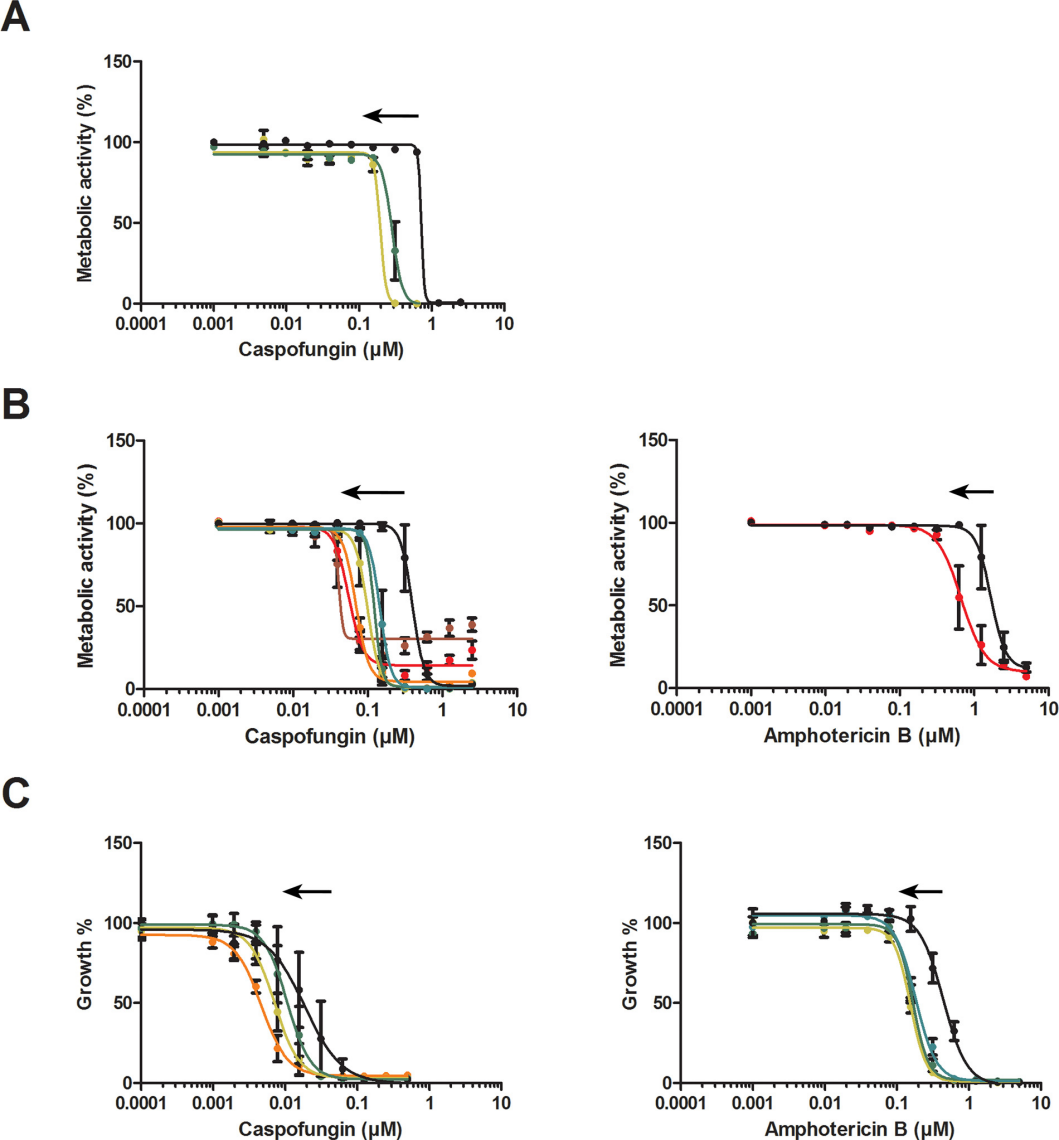
Synergy between rHsAFP1 and caspofungin or amphotericin B, for (A) biofilm inhibition, as determined by CTB assay; (B) biofilm eradication, as determined by CTB assay; and (C) growth inhibition of planktonic cultures. Growth was analysed by measuring the OD_490_. Sigmoidal curves were generated using data of at least three independent experiments (n ≥ 3), using the model Y = Bottom+(Top-Bottom)/(1+10^((LogIC50-X)*HillSlope)) in GraphPad Prism. Dose response curves of caspofungin in the presence of synergistic concentrations of rHsAFP1 are presented. Black arrows represent synergy. Coloured lines represent different rHsAFP1 doses, as follows: brown: 16.8 μM; red: 8.4 μM; orange: 4.2 μM; dark yellow: 2.1 μM; green: 1.05 μM; turquois: 0.53 μM; blue: 0.26 μM and black: 0 μM.

As rHsAFP1 prevented *C*. *albicans* biofilm formation, we further investigated the effect of rHsAFP1 on the biofilm inhibitory and eradicating activity of conventional antimycotics, such as caspofungin and amphotericin B. To this end, checkerboard assays were performed and the corresponding FICI values were calculated to determine whether rHsAFP1 acts synergistically with these compounds against *C*. *albicans* biofilms (Fig 5, Tables 1 and 2).

In the biofilm inhibition assays (Table 1), synergistic effects (FICI ≤ 0.5) were observed between rHsAFP1 and caspofungin: rHsAFP1 increased the activity of caspofungin at concentrations of 1.05 μM and 2.1 μM, resulting in a 2.5-fold and 3.7-fold reduction of the caspofungin BIC50, respectively. Although not synergistic, 0.53 μM, 4.2 μM and 8.4 μM rHsAFP1 also reduced the BIC50 of caspofungin significantly (*P*<0.05). No synergistic effects were observed between rHsAFP1 and amphotericin B in the biofilm inhibition assays, however, a range of 1.05 μM to 8.4 μM rHsAFP1 significantly reduced the amphotericin B BIC50. Moreover, we also found that rHsAFP1 acted synergistically with caspofungin or amphotericin B in the eradication of *C*. *albicans* biofilms (Table 2): all rHsAFP1 concentrations tested (*i*.*e*. a range from 0.53 μM to 16.8 μM rHsAFP1) increased the biofilm eradicating capacity of caspofungin and although only 8.4 μM rHsAFP1 displayed synergy with amphotericin B, multiple concentrations significantly reduced the BEC50 of amphotericin B.

To assess whether the synergistic effects observed between rHsAFP1 and amphotericin B or caspofungin against *C*. *albicans* biofilms were biofilm-specific, a similar checkerboard assay was performed on planktonic *C*. *albicans* cells (Table 3). Synergistic effects were observed between rHsAFP1 and caspofungin or amphotericin B against planktonic *C*. *albicans* cells and hence, synergy between rHsAFP1 and these compounds seems not biofilm-specific. Synergy between rHsAFP1 and amphotericin B was observed at lower rHsAFP1 concentrations as compared to those observed between rHsAFP1 and caspofungin. Interestingly, the concentration range of rHsAFP1 that acted synergistically with caspofungin against planktonic *C*. *albicans* cells was more restricted as compared to a *C*. *albicans* biofilm setup: all rHsAFP1 concentrations tested (*i*.*e*. 0.53 μM to 16.8 μM) increased caspofungin activity against *C*. *albicans* biofilms in the biofilm eradication assays, whereas only 2.1 μM and 4.2 μM rHsAFP1 acted synergistically with caspofungin against planktonic *C*. *albicans* cells. In addition, only 1.05 μM and 2.1 μM rHsAFP1 enhanced caspofungin activity against *C*. *albicans* biofilms in the biofilm inhibition assays. This indicates that synergy between caspofungin and rHsAFP1 is more evident in the eradication of *C*. *albicans* biofilms. In contrast, synergy between amphotericin B and rHsAFP1 was more pronounced against planktonic *C*. *albicans* cultures, as various rHsAFP1 concentrations (*i*.*e*. 0.53 μM to 2.1 μM) acted synergistically with amphotericin B against planktonic *C*. *albicans* cells and only 8.4 μM rHsAFP1 increased amphotericin B activity against *C*. *albicans* biofilms in the biofilm eradication assay. No synergistic effects between amphotericin B and rHsAFP1 were observed in the biofilm inhibition assays.

### rHsAFP1 does not affect HepG2 cell viability and proliferation

Various plant defensins are reported to be non-toxic to human cells due to their fungal membrane-specific interactions [11]. As no records exist yet on potential toxicity of HsAFP1, we analysed the effect of rHsAFP1 on human hepatoma cells (HepG2) and found that rHsAFP1 did not affect HepG2 cell viability nor cell proliferation up to 40 μM, the highest rHsAFP1 concentration tested in this setup. No statistically significant differences were found in cell viability and proliferation between untreated and rHsAFP1-treated cells (S1 Fig).

### The γ-core and adjacent regions are important for rHsAFP1 antibiofilm activity

**Fig 6:**
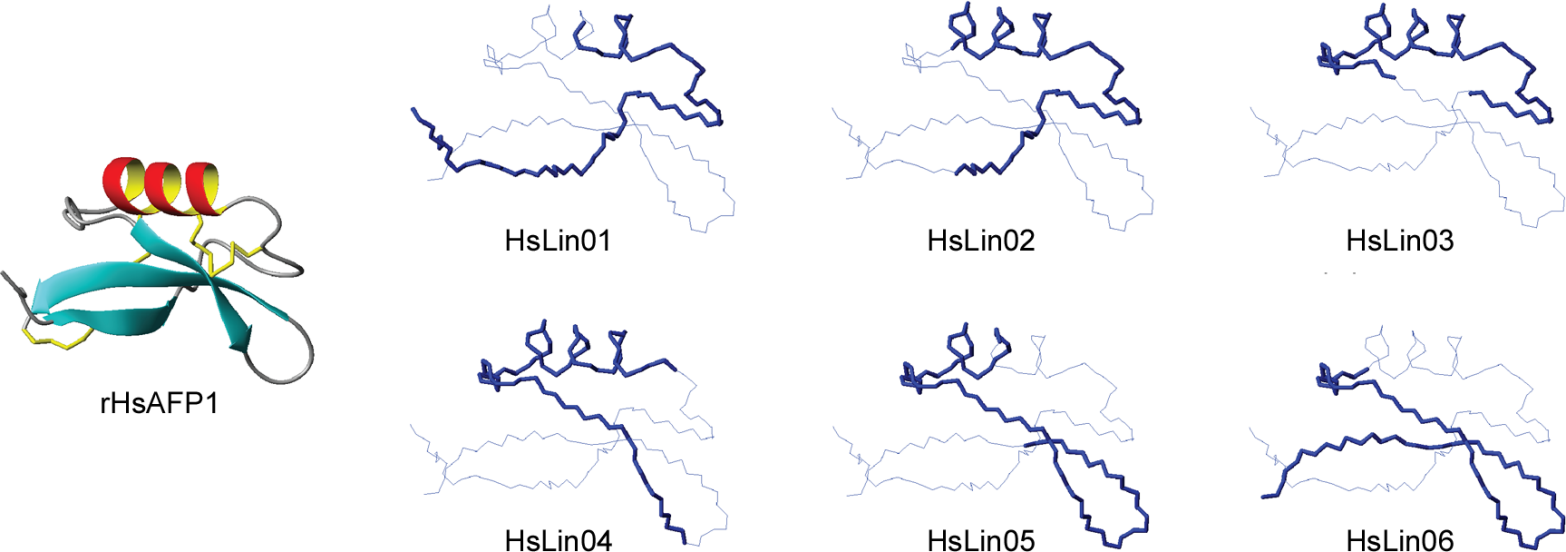
Representation of the HsLin peptides imposed on the rHsAFP1 structure, according to the amino acid sequence. HsLin peptides are shown as a thick blue line in the same orientation as rHsAFP1; other residues of rHsAFP1, not present in the HsLin peptide, are shown as a thin blue line. Note that (i) the cysteine residues are replaced by α-aminobutyric acid to avoid formation of disulfide bonds and that (ii) the CSαβ scaffold is not present in the HsLin peptides, and therefore, the peptides do not adopt the same conformation as the mature rHsAFP1.

In order to gain insights in the structure-function relationship of HsAFP1 against *C*. *albicans* planktonic and biofilm cells, we conducted a structure-function relationship study using HsAFP1-derived linear fragments. The selection of fragments was based on the procedure used by Schaaper *et al*. [68]. We synthesized 24-mer peptides with an 18-mer overlap, spanning the entire HsAFP1 amino acid sequence and analysed these peptides for their activity towards *F*. *culmorum* and *C*. *albicans* planktonic cultures and biofilms. The sequences of the linear fragments (HsLin01-HsLin06) are presented in Fig 1B. Fig 6 shows a diagram in which the HsLin peptides are imposed on the rHsAFP1 structure, according to their amino acid sequence. Note that (i) the cysteine residues are replaced by α-aminobutyric acid to avoid formation of disulfide bonds and that (ii) the CSαβ scaffold is not present in the HsLin peptides, and therefore, the peptides do not adopt the same conformation as the mature rHsAFP1.

**Fig 7:**
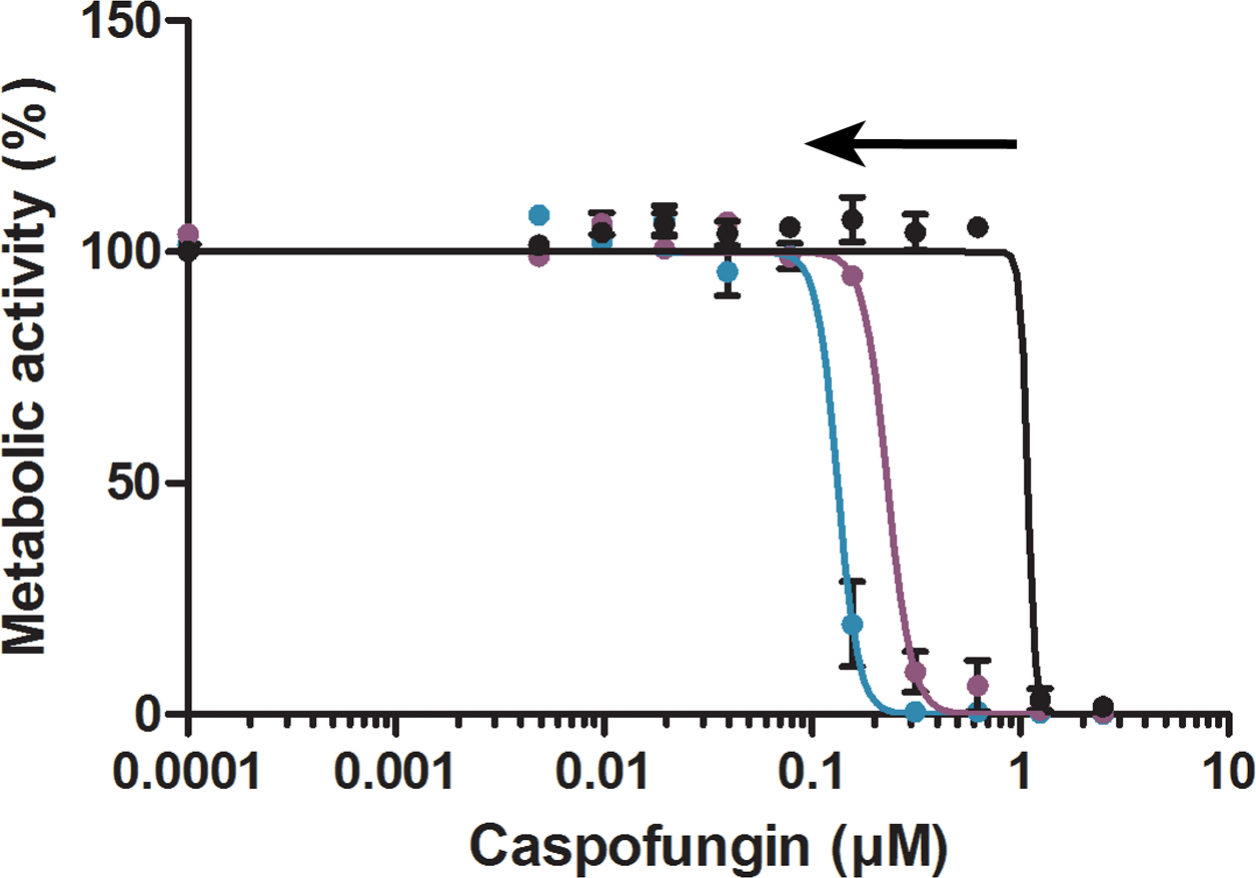
Synergy between caspofungin and HsLin06 for biofilm inhibition. Metabolic activity was measured using CTB. Sigmoidal curves were generated using data of at least three independent experiments (n ≥ 3), using the model Y = Bottom+(Top-Bottom)/(1+10^((LogIC50-X)*HillSlope)) in GraphPad Prism. Dose response curves of caspofungin in the presence of synergistic concentrations of HsLin06 are presented. Black arrows represent synergy. Coloured lines represent different HsLin doses, as follows: brown: 43.75 μM; orange: 21.88 μM; dark yellow: 10.94 μM; green: 5.47 μM; blue: 1.5; purple: 0.75 μM μM and black: 0 μM.

None of the linear HsAFP1-derived fragments inhibited the growth of *F*. *culmorum* up to the highest tested concentration, 1.5 μM, whereas rHsAFP1 inhibited growth of this fungus with an IC50 value of 0.45 ± 0.13 μM. In addition, these truncated peptides did not inhibit the growth of *C*. *albicans* in contrast to full-length rHsAFP1. Hundred percent growth inhibition of *C*. *albicans* planktonic cells was observed at 70 μM for rHsAFP1, whereas concentrations up to 350 μM of the peptides were not sufficient to cause 100% growth inhibition. Furthermore, only HsLin06 inhibited *C*. *albicans* biofilm formation to the same extent as rHsAFP1: the BIC50 values of HsLin06 and rHsAFP1 were 10.80 ± 3.59 μM and 11.00 ± 1.70 μM, respectively (Table 5), suggesting that the sequence comprising HsLin06 is important for antibiofilm activity. HsLin03 and HsLin05 showed antibiofilm activity as well, however, with a 10- to 15-fold higher BIC50 value than that of rHsAFP1 or HsLin06. Other fragments did not inhibit biofilm formation up to 175 μM, the highest tested concentration. We further analysed the potential of the peptides to increase the activity of caspofungin to prevent biofilm formation. We found that HsLin06, but also HsLin01 and HsLin05, acted synergistically with caspofungin to inhibit *C*. *albicans* biofilm formation in a range of 0.75 μM to 1.5 μM (Fig 7 and Table 5 for HsLin06 and S2 Fig for the other HsLin). We did not observe synergistic effects between the other linear fragments and caspofungin for preventing biofilm formation (S2 Fig).

**Table 5:** Structure-function relationship study of HsAFP1-derived fragments against *C*. *albicans* biofilms*.

Peptide	BIC50 (μM) ± SEM	Significance level
rHsAFP1	11.00 ± 1.70	
HsLin01	>175	***
HsLin02	>175	***
HsLin03	96.78 ± 15.90	**
HsLin04	>175	***
HsLin05	160.00 ± 33.36	*
HsLin06	10.80 ± 3.59	NS

* BIC50 values were determined by CTB assay; mean ± SEM for n ≥ 3 independent experiments is presented; BIC50, minimum inhibitory concentration that is required to inhibit biofilm formation by 50% as compared to control treatment. Unpaired Student t-tests were performed to analyse significant differences between the effect of the linear fragments and rHsAFP1; the significance level is presented (*, ** and *** represent *P*<0.05, *P*<0.01 and *P*<0.001, respectively; NS, no significant difference).

## Discussion

We have generated recombinant (r) HsAFP1, a plant defensin from the seeds of coral bells (*Heuchera sanguinea)* [32], in *Pichia pastoris* with a yield of at least 40 mg/L of culture of purified peptide. The recombinant peptide rHsAFP1 was characterized by potent antifungal activity, similar to that of HsAFP1 purified from seeds. NMR analysis revealed that rHsAFP1 adopts the characteristic cysteine-stabilised αβ-motif, similar to other plant defensins [2,4–6]. The NMR results, together with the results of the antifungal activity assays, led us to conclude that *P*. *pastoris* is an ideal heterologous production system for plant defensins as highly structured and active peptides were obtained, without affecting their antifungal activity.

Plant defensins might be of interest in the development of novel antimycotics, as they are in general non-toxic towards human cells [11–13] and there is a strong need for novel agents to combat fungal infections. The latter is of great importance in fungal biofilm-related infections, as only few compounds can be used to treat these diseases [29–31]. It was already shown that the plant defensin HsAFP1 is characterized by potent antifungal activity towards *C*. *albicans* [33], and more interestingly, by a low *in vitro* frequency of resistance occurrence in planktonic *C*. *albicans* cultures [11]. Hence, we investigated the potential antibiofilm activity of rHsAFP1 against *C*. *albicans* biofilms. rHsAFP1 prevented *C*. *albicans* biofilm formation, resulting in a BIC50 value of 11.00 ± 1.70 μM, whereas the peptide was not able to eradicate *C*. *albicans* biofilms. SEM images of *C*. *albicans* biofilms indicated that control biofilms were able to form a dense hyphal network within four hours after adhesion to the surface, whereas biofilms grown in the presence of rHsAFP1 mainly consisted of cells attached to the surface without formation of a hyphal network. It needs to be further investigated whether this observation is due to inhibition of the yeast-to-hypha transition by rHsAFP1. Note that the latter has been previously reported for the plant defensin RsAFP2 in planktonic *C*. *albicans* cultures [22], and might indicate a similar mechanism of action for RsAFP2 and rHsAFP1, although different fungal membrane targets might be involved [33]. Checkerboard assays revealed that, although all tested concentrations of rHsAFP1 acted synergistically with caspofungin in eradication of *C*. *albicans* biofilms, only specific rHsAFP1 doses proved synergistic with caspofungin in inhibiting *C*. *albicans* biofilm or planktonic cell growth. The underlying molecular mechanism resulting in the improved activity of rHsAFP1 in combination with caspofungin for eradicating biofilms is not clear. In case of amphotericin B, most pronounced synergies with rHsAFP1 were apparent against planktonic *C*. *albicans* cells, although still in a rather limited rHsAFP1 concentration range. We found that rHsAFP1 did not affect the cell viability and cell proliferation of human hepatoma cells (HepG2) up to 40 μM, the highest concentration tested in this setup, suggesting that rHsAFP1 is not toxic to human cells. This is in line with previous reports on the non-toxicity of plant defensins towards human cells [11–13].

A structure-function relationship study with 24-mer peptides spanning the entire HsAFP1 amino acid sequence showed that the γ-core and its adjacent regions are important for antibiofilm activity, as only HsLin06 had a similar antibiofilm activity to that of rHsAFP1. In addition, we found that antifungal and antibiofilm activity of rHsAFP1 against *C*. *albicans* are probably not linked, as HsLin06 inhibited biofilm formation to the same extent as rHsAFP1 without inhibiting planktonic growth. Checkerboard analyses revealed that HsLin01, HsLin05 and HsLin06 acted synergistically with caspofungin in the prevention of *C*. *albicans* biofilm formation. Hence, it seems that antibiofilm activity is not essential to increase the activity of caspofungin against *C*. *albicans* biofilms, indicating that antibiofilm activity and the ability to cause synergistic effects with caspofungin are not linked. Synergy between caspofungin and other compounds, including toremifene citrate, tyrocidines, posaconazole, cefoperazone-sulbactam (CPZ/SAM), piperacillin-tazobactam (PIP/TAZ) and colistin, against *Candida* biofilms has been described before [54,69–71] and might point to a general effect of caspofungin against fungal biofilms. In this respect, we recently identified a biofilm-specific enhancement of caspofungin activity by toremifene citrate against *C*. *albicans* and *C*. *glabrata* biofilms, resulting in up to 20-fold reduction of the caspofungin BIC50 [54]. Similarly, it was reported that CPZ/SAM and PIP/TAZ enhance caspofungin activity *in vitro* and *in vivo* against *C*. *albicans*. In that study, CPZ/SAM is suggested to have more affinity for the same efflux pump as caspofungin, leading to an increase in intracellular levels of caspofungin and hence, synergy between caspofungin and CPZ/SAM [70]. In another report, Chen and colleagues demonstrated that posaconazole exhibits synergistic antifungal activity with caspofungin *in vitro* and *in vivo* against *C*. *albicans* [71]. In addition, it was reported that tyrocidines exhibit a pronounced synergistic biofilm-eradicating activity in combination with caspofungin and amphotericin B against *C*. *albicans* biofilms [69]. In the latter study, a more pronounced synergy between tyrocidines and caspofungin was observed as compared to amphotericin B, and, as amphotericin B and tyrocidines both target cell membranes, it was hypothesized that the observed effect was due to competition for this target. This hypothesis might also be valid for our observations, as plant defensins specifically target the fungal membrane [18] and a higher synergy between rHsAFP1 and caspofungin was observed as compared to rHsAFP1 and amphotericin B.

Finally, Zeidler and co-workers reported synergy between echinocandins and colistin against *Candida* spp. They suggested that this synergy is a result of echinocandin-mediated weakening of the cell wall that leads to facilitated colistin-targeting of fungal membranes, which in turn reinforces the antifungal activity of echinocandins [72]. Whether this is the case for rHsAFP1, needs to be further investigated.

This study is the first to report the activity of a plant defensin towards fungal biofilms *in vitro* and indicates, together with other reports on the antifungal and/or antibiofilm activity of human and insect defensins [73–80], the relevance of using defensins as an approach to combat fungal biofilm-associated infections. We showed that rHsAFP1 inhibited *C*. *albicans* planktonic growth and biofilm formation, and did not affect the viability and proliferation of human HepG2 cells *in vitro*. The latter indicates that HsAFP1 does not exhibit a general cytotoxicity, which is supported by its lack of inhibitory activity to bacteria [32]. It was already shown that the plant defensin RsAFP2 is prophylactically effective against murine candidiasis [81], pointing to the *in vivo* potential of plant defensins. Moreover, we showed that rHsAFP1 acted synergistically with caspofungin against *C*. *albicans* biofilms and planktonic cells. In addition, we found that certain linear HsAFP1-derived fragments also increased the activity of caspofungin to prevent biofilm formation. A combinatorial approach to combat fungal infections is often more effective and decreases the chance of resistance occurrence [34]. Our results indicate a potentiating effect of rHsAFP1 and its derivatives on caspofungin, which should be further investigated *in vivo*. Taken together, rHsAFP1 and its derivatives are interesting peptides for further development as an antifungal or antibiofilm agent for use alone or in a multi-drug approach to combat fungal infections.

## Supporting Information

S1 Table ^1^H assignments for rHsAFP1 in 10% D_2_O/90% H_2_O, pH 4.0 at 298 K. (PDF)

S2 Table Statistical analysis of rHsAFP1 structures. (PDF)

S1 Fig rHsAFP1 does not affect HepG2 cell viability and cell proliferation. HepG2 cells were treated with water (control treatment) or rHsAFP1 (0.01 μM– 42 μM) for 24 hours. Cell viability and cell proliferation were determined by XTT staining and BrdU staining, respectively, and results were expressed relative to cells receiving control treatment. Mean and SEM of three experiments in quadruplicate is shown. No statistically significant differences were found in cell viability and cell proliferation between untreated (control treatment) and rHsAFP1-treated cells up to the highest tested rHsAFP1 concentration (*i*.*e*. 40 μM) (Unpaired Student t-test; *P*<0.05 was defined as statistically significant).
(TIF)

S2 Fig Synergy between caspofungin and HsLin01 (A), HsLin02 (B), HsLin03 (C), HsLin04 (D) and HsLin05 (E) for biofilm inhibition. Metabolic activity is measured using CTB. Sigmoidal curves were generated using data of at least three independent experiments (n ≥ 3), using the model Y = Bottom+(Top-Bottom)/(1+10^((LogIC50-X)*HillSlope)) in GraphPad Prism. Dose response curves of caspofungin in the presence of synergistic concentrations of HsLin are presented. Black arrows represent synergy. Coloured lines represent different HsLin doses, as follows: brown: 43.75 μM; orange: 21.88 μM; dark yellow: 10.94 μM; green: 5.47 μM; blue: 1.5; purple: 0.75 μM μM and black: 0 μM.
(TIF)
