# Supplementary material for: Synergistic Activity of the Plant Defensin HsAFP1 and Caspofungin against Candida albicans Biofilms and Planktonic Cultures
Source: PLoS One. 2015 Aug 6;10(8):e0132701. doi: 10.1371/journal.pone.0132701 (PMC4527839; doi:10.1371/journal.pone.0132701)
Supplement: S1 Table — (PDF) [file pone.0132701.s001.pdf]

S1 Table ^{1}H assignments for rHsAFP1 in 10% D_2O /90% H_2O, pH 4.0 at 298 K.

Residue	Chemical shifts			others
	NH	αH	βH	
Asp1				
Gly2	8.64	4.03, 4.01		
Val3	8.03	4.05	1.91	γCH_3 0.83, 0.74
Lys4	8.55	4.27	1.80, 1.80	γCH_2 1.36, 1.35; δCH_2 1.65, 1.65; εCH_2 2.96, 2.96; ζNH_3^+ 7.49
Leu5	8.20	4.99	1.58, 0.90	γCH 1.29; δCH_3 0.58, 0.00
Cys6	9.41	4.91	2.83, 2.75	
Asp7	8.50	5.29	2.40, 2.19	
Val8	9.34	4.78	1.87	γCH_3 0.82, 0.72
Pro9		3.74	1.95, 1.75	γCH_2 2.12, 1.89; δCH_2 3.87, 3.72
Ser10	7.95	4.53	3.93, 3.53	
Gly11	10.29	4.29, 3.87		
Thr12	8.18	4.49	4.12	γCH_3 1.07
Trp13	8.07	4.16	3.17, 3.08	δ1CH 7.05; ε1NH 8.75; ε3CH 7.39; η2CH 7.17; ζ2CH 7.77; ζ3CH 6.85
Ser14	7.44	4.58	3.57, 3.55	
Gly15	8.33	4.07, 3.88		
His16	8.82	4.53	3.32, 2.23	δCH 7.33; εCH 8.66
Cys17	8.29	4.74	2.90, 1.87	
Gly18	8.50	4.26, 3.85		
Ser19	7.23	4.83	4.04, 3.78	
Ser20	9.48	4.20	4.05, 4.05	
Ser21	8.52	4.27	3.87, 3.87	
Lys22	7.91	4.26	2.19, 2.08	γCH_2 1.72, 1.62; δCH_2 1.85, 1.84; εCH_2 3.04, 3.04; ζNH_3^+ 7.60
Cys23	7.60	4.23	2.32, 2.32	
Ser24	8.53	4.28	4.15, 3.68	
Gln25	8.06	3.99	2.26, 2.26	γCH_2 2.57, 2.45; εNH_2 7.68, 6.91
Gln26	8.25	4.18	2.54, 2.41	γCH_2 2.53, 2.45; εNH_2 7.87, 6.65
Cys27	8.80	4.33	3.13, 3.13	
Lys28	8.33	4.35	1.82, 1.82	γCH_2 1.48, 1.48; δCH_2 1.63, 1.63; εCH_2 2.92, 2.92; ζNH_3^+ 7.54
Asp29	9.02	4.49	2.90, 2.84	
Arg30	8.34	4.49	2.03, 2.03	γCH_2 1.77, 1.67; δCH_2 3.19, 3.19; εNH 7.36
Glu31	6.90	4.39	2.28, 2.23	γCH_2 2.75, 2.68
His32	6.84	4.40	3.35, 3.35	δCH 7.12; εCH 8.57
Phe33	8.61	4.88	3.54, 2.69	δCH 7.45; εCH 7.38
Ala34	8.57	4.00	1.26	
Tyr35	9.08	4.34	3.14, 2.73	δCH 7.30; εCH 6.83
Gly36	8.23	4.18, 3.60		
Gly37	7.89	4.57, 4.38		
Ala38	9.06	4.30	1.39	
Cys39	8.38	5.45	2.66, 2.51	
His40	9.48	4.87	3.04, 3.04	δCH 6.72; εCH 8.31
Tyr41	9.05	4.53	3.02, 2.84	δCH 6.98; εCH 6.78
Gln42	8.17	4.09	1.95, 1.95	γCH_2 2.34, 2.19; εNH_2 7.75, 6.97
Phe43	8.80	4.10	3.26, 2.89	δCH 7.38; εCH 7.38
Pro44		3.95	1.98, 0.67	γCH_2 1.66, 1.47; δCH_2 3.29, 3.28
Ser45	8.39	4.95	3.70, 3.68	
Val46	8.55	4.34	1.92	γCH_3 1.14, 0.90
Lys47	8.35	4.56	1.68, 1.42	γCH_2 1.35, 1.25; δCH_2 1.55, 1.55; εCH_2 2.91, 2.91; ζNH_3^+ 7.54
Cys48	8.87	4.65	2.15, 1.69	
Phe49	8.53	4.78	2.80, 2.56	δCH 6.82; εCH 7.21
Cys50	9.00	5.37	2.45, 1.39	
Lys51	8.21	5.00	1.37, 1.37	γCH_2 1.12, 1.13; δCH_2 1.54, 1.38; εCH_2 2.77, 2.77; ζNH_3^+ 7.55
Arg52	8.84	4.88	1.98, 1.85	γCH_2 1.59, 1.56; δCH_2 3.23, 3.21; εNH 7.20
Gln53	9.04	4.54	2.16, 2.05	γCH_2 2.43, 2.43; εNH_2 7.70, 7.07
Cys54	8.51	4.57	3.16, 3.16	
