# Supplementary material for: Synergistic Activity of the Plant Defensin HsAFP1 and Caspofungin against Candida albicans Biofilms and Planktonic Cultures
Source: PLoS One. 2015 Aug 6;10(8):e0132701. doi: 10.1371/journal.pone.0132701 (PMC4527839; doi:10.1371/journal.pone.0132701)
Supplement: S2 Table — (PDF) [file pone.0132701.s002.pdf]

S2 Table Statistical analysis of rHsAFP1 structures*

Experimental restraints	
Total no. distance restraints	602
Intraresidue	162
Sequential	173
Medium range, *i-j*<5	66
Long range, *i-j*≥5	201
Hydrogen bond restraints	30
Disulphide bond restraints	24
Dihedral angle restraints	
Phi	45
Psi	37
Chi1	19
Total number of restraints per residue	14.0
Rms deviation from mean structure, Å	
Backbone atoms	1.04 ± 0.24
All heavy atoms	1.66 ± 0.24
Stereochemical quality[a]	
Residues in most favoured Ramachandran region, %	85.5 ± 3.1
Ramachandran outliers, %	1.5 ± 1.8
Unfavourable sidechain rotamers, %	9.0 ± 5.7
Clashscore, all atoms	2.4 ± 1.4
Overall MolProbity score	2.27 ± 0.18

*All statistics are given as mean ± SD.

[a]According to MolProbity [1].

References

1. Chen VB, Arendall WB, 3rd, Headd JJ, Keedy DA, Immormino RM, et al. (2010) MolProbity: all-atom structure validation for macromolecular crystallography. Acta Crystallogr D Biol Crystallogr 66: 12-21.
