# Supplementary figures and images for: Synergistic Activity of the Plant Defensin HsAFP1 and Caspofungin against Candida albicans Biofilms and Planktonic Cultures
Source: PLoS One. 2015 Aug 6;10(8):e0132701. doi: 10.1371/journal.pone.0132701 (PMC4527839; doi:10.1371/journal.pone.0132701)

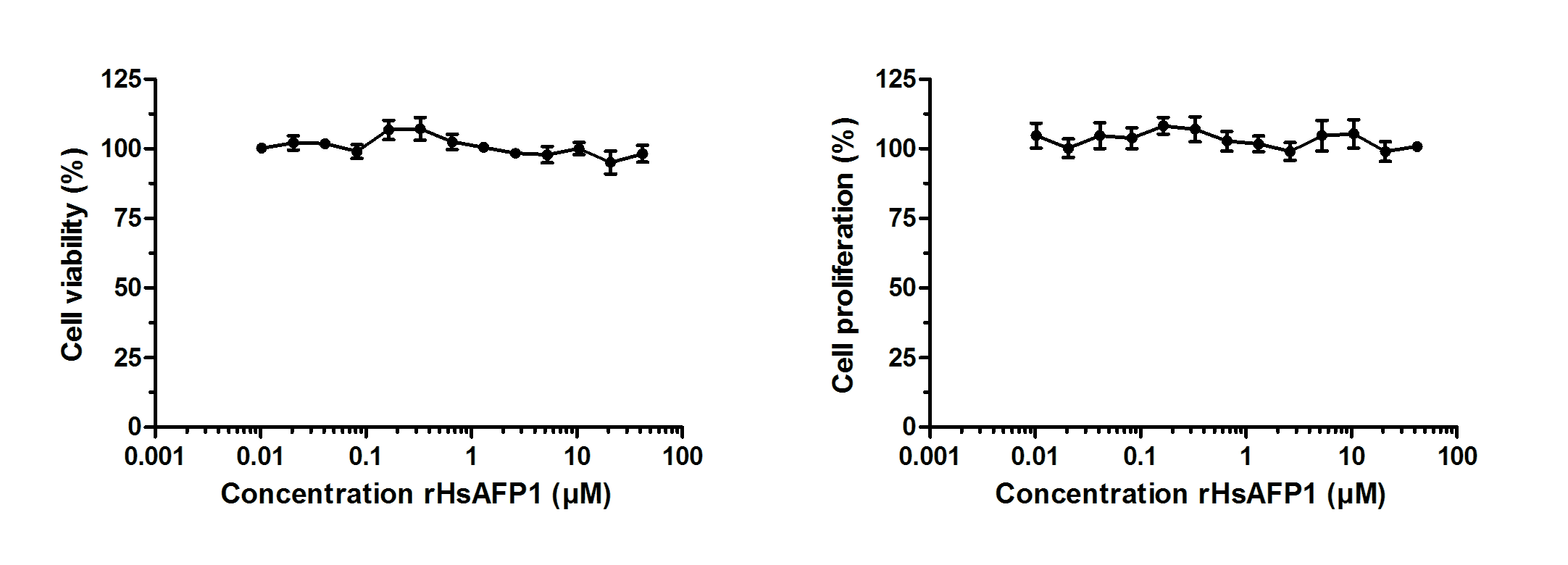

Supplement: S1 Fig — HepG2 cells were treated with water (control treatment) or rHsAFP1 (0.01 μM– 42 μM) for 24 hours. Cell viability and cell proliferation were determined by XTT staining and BrdU staining, respectively, and results were expressed relative to cells receiving control treatment. Mean and SEM of three experiments in quadruplicate is shown. No statistically significant differences were found in cell viability and cell proliferation between untreated (control treatment) and rHsAFP1-treated cells up to the highest tested rHsAFP1 concentration (i.e. 40 μM) (Unpaired Student t-test; P<0.05 was defined as statistically significant). (TIF) [file pone.0132701.s003.tif]

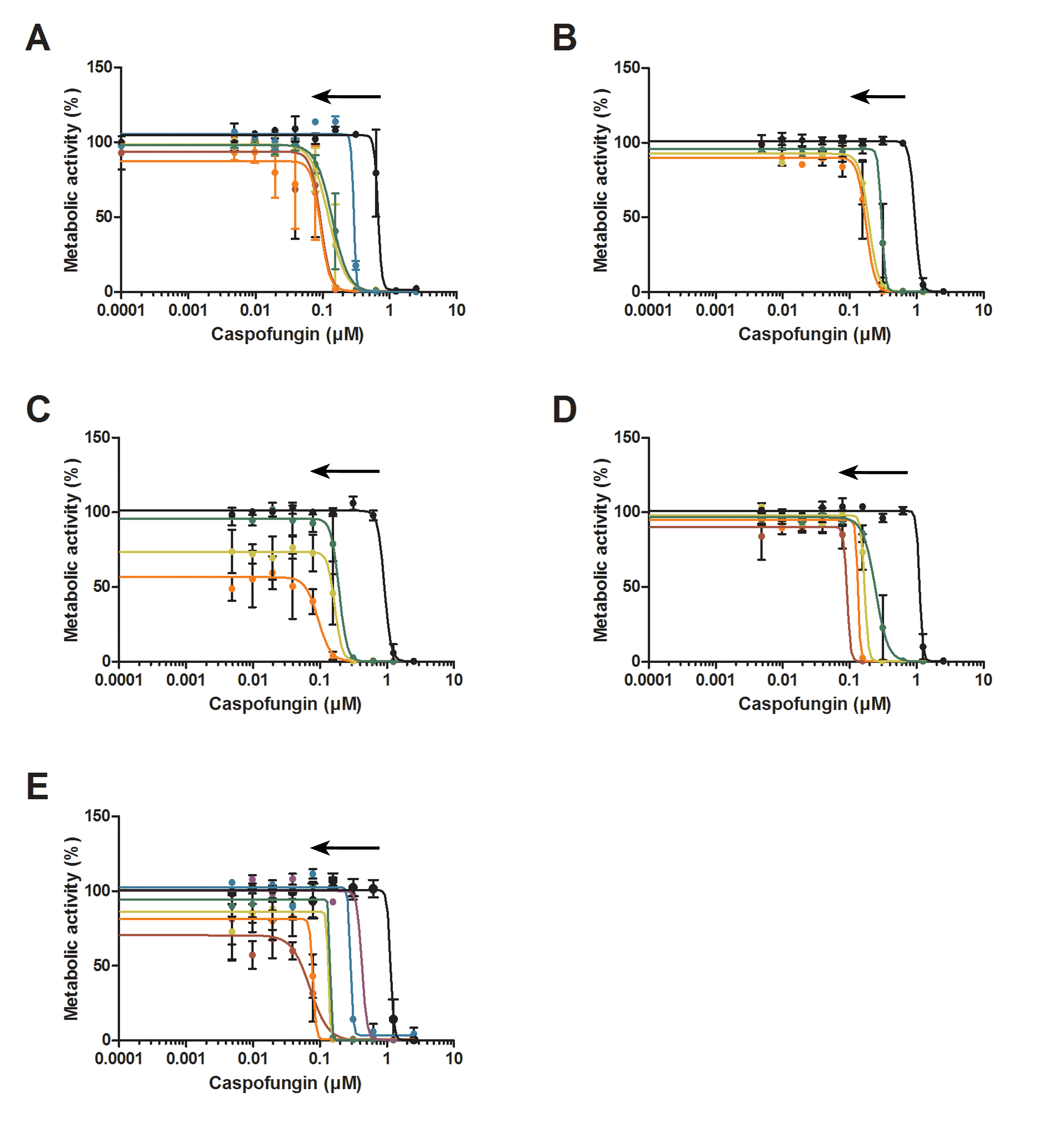

Supplement: S2 Fig — Metabolic activity is measured using CTB. Sigmoidal curves were generated using data of at least three independent experiments (n ≥ 3), using the model Y = Bottom+(Top-Bottom)/(1+10^((LogIC50-X)*HillSlope)) in GraphPad Prism. Dose response curves of caspofungin in the presence of synergistic concentrations of HsLin are presented. Black arrows represent synergy. Coloured lines represent different HsLin doses, as follows: brown: 43.75 μM; orange: 21.88 μM; dark yellow: 10.94 μM; green: 5.47 μM; blue: 1.5; purple: 0.75 μM μM and black: 0 μM. (TIF) [file pone.0132701.s004.tif]
